# Endoscopic Dilation of Refractory Postlaryngectomy Strictures: A Case Series and Literature Review

**DOI:** 10.1155/2019/8905615

**Published:** 2019-05-28

**Authors:** Patrick L. Stoner, Amy L. Fullerton, Alyssa M. Freeman, Neil N. Chheda, David S. Estores

**Affiliations:** ^1^Medicine, University of Florida, 1600 SW Archer Rd, Gainesville, FL 32610, USA; ^2^Speech, Language, and Hearing Sciences, University of Florida, 1600 SW Archer Rd, Gainesville, FL 32610, USA; ^3^Otolaryngology, University of Florida, 1600 SW Archer Rd, Gainesville, FL 32610, USA; ^4^Gastroenterology, Hepatology & Nutrition, University of Florida, 1600 SW Archer Rd, Gainesville, FL 32610, USA

## Abstract

**Background:**

Endoscopic dilation of postlaryngectomy strictures (PLS) is safe and effective; however, PLS are often refractory and require serial dilations. Long-term outcomes of dilation in patients with refractory PLS are not well reported.

**Materials and Methods:**

Seven patients with dysphagia and refractory PLS underwent serial endoscopic dilation therapy during a 4.5-year period. Dilation characteristics, technical success, clinical success, and diet advancement (as assessed by Diet/GT scale) were measured*. Results*. All strictures were complex, and there were no reported complications. The median number of dilations per patient was 12 (range 7 to 48). The average interval in between dilations was six weeks. Technical success was achieved in 6/7 patients, and clinical success was achieved in 2/7 patients. 6/7 patients had advancements in Diet/GT scores.

**Conclusions:**

Dilation of refractory PLS is safe and effective and frequently requires the use of a retrograde approach, fluoroscopic guidance, and/or lumen patency strings. Serial dilations are required to maintain luminal patency, relieve dysphagia, and advance oral diet. The definition of clinical success of dilation in these patients should avoid the use of a specific time interval between dilations to characterize success.

## 1. Introduction

Dysphagia and pharyngoesophageal strictures are frequent complications associated with treatment for head and neck cancer and can negatively affect quality of life and lead to social isolation [1]. Laryngectomy, radiation (in a dose-volume relationship, in particular intensity-modulated radiation therapy (IMRT)), radiation in combination with chemotherapy, and chemoradiotherapy plus surgery have all been shown to lead to pharyngoesophageal stricture formation [1–6]. Dysphagia following laryngectomy has a reported incidence ranging between 16 and 72% and in roughly one-third to one-half of these cases; a benign stricture is determined to be the cause of dysphagia [1, 7, 8]. A reduction in amplitude and duration of pharyngeal peristalsis and a decrease in pharyngeal sensation as a result of sectioning of the superior laryngeal nerve and recurrent laryngeal nerve can also lead to dysphagia after laryngectomy [8]. Xerostomia and neuromuscular dysfunction attributed to concurrent radiotherapy can also lead to dysphagia [2]. Stricture formation may be due to collagen deposition and fibrin production from deep ulceration or chronic inflammation. In this setting, recurrent and/or refractory anastomotic strictures result from cicatricial luminal compromise or fibrosis in the absence of inflammation on endoscopy [9, 10].

Several studies [4, 5, 7, 9, 11, 12] have shown that endoscopic dilation of pharyngoesophageal strictures that develop after head and neck cancer treatment is generally safe and effective; however, these strictures are often refractory and require serial dilations. Strictures are considered refractory when luminal patency of ≥14 mm is not achieved after ≥5 dilation sessions at intervals of two to four weeks [10]. Refractory postlaryngectomy strictures (PLS) have not been well characterized, and outcomes of dilation in these patients have not been well reported. In addition, while surgical factors associated with recurrent or refractory anastomotic strictures have been described [1, 9], studies investigating clinical and endoscopic factors are lacking. We describe characteristics of this patient population and report outcomes of a single endoscopist's experience in treating refractory PLS. We also review the available literature and propose modifications in the way therapeutic success is measured in treatment of these refractory strictures.

## 2. Materials and Methods

For this retrospective cohort study, we queried our institution's Integrated Data Repository (IDR) to identify patients with prior history of laryngectomy who had documented clinical dysphagia treated with endoscopic dilation therapy from May 2013 to September 2017. Only patients with refractory (defined below) postlaryngectomy strictures (PLS) and a minimum of five dilations were included. All patients had at least one month of follow-up after dilation. For this study, all dilations meeting criteria were performed by a single experienced endoscopist (D.E.). In total, there were 118 dilations performed, with a median follow-up period of 11 months (average follow-up period, 17.6 months). The institutional review board approved this protocol.

### 2.1. Patients

In total, seven patients (four men and three women, five Caucasian and two African-American, average age 60 years) were included. All seven patients underwent total laryngectomy (TL) as a result of SCC of the larynx. All patients had prior alcohol use, and six of seven patients had prior tobacco use. All patients had received radiation (three after TL, three before TL, and one both before and after TL). Five of the seven patients had received chemotherapy (three after TL and two before TL). All strictures were pharyngoesophageal, ranging from 10 to 17 cm from the incisors. Six of seven patients had a tracheoesophageal voice prosthesis (TEP) in place. Patient 1 had GT placed preoperatively for alimentation, whereas patients 2, 3, and 4 had GT placed postoperatively for alimentation. Patient 6 initially had GT placed for postoperative percutaneous fistula, but continued to require a GT for alimentation due to severe lumen stenosis (initial dilation found complete lumen occlusion). See Table 1 for patient's characteristics (including each patients' malignancy treatment) and TL characteristics.

### 2.2. Strictures and Dilations

**Figure 1:**
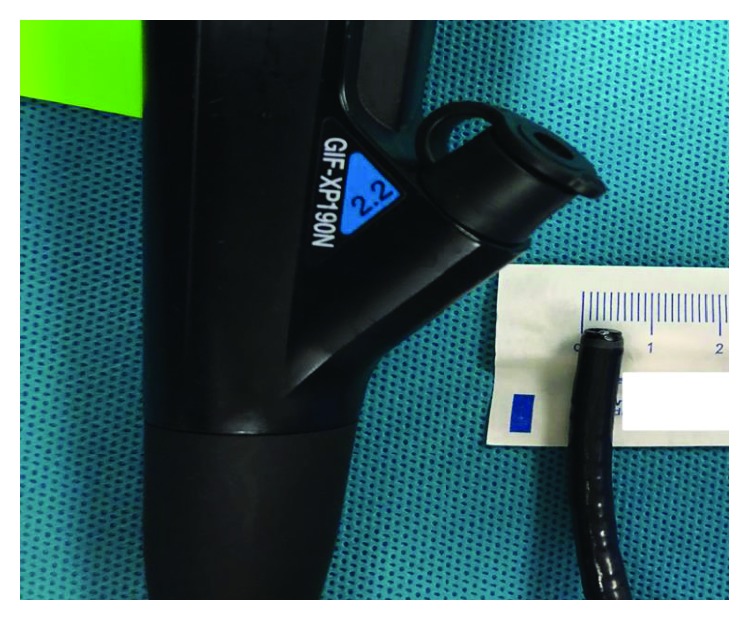
Olympus® GIF-XP190N endoscope measuring 5.5 mm.

**Figure 2:**
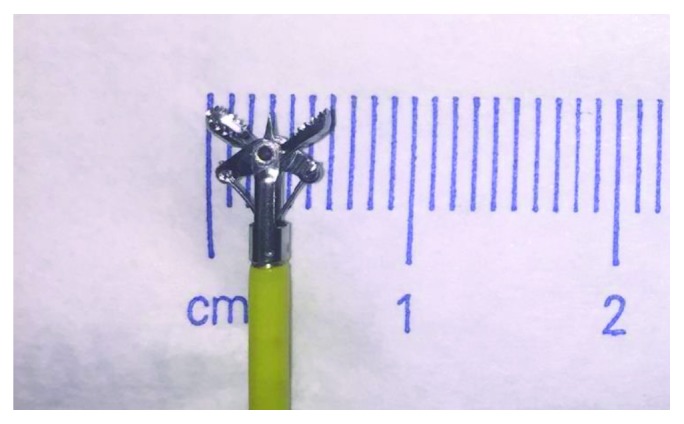
Pediatric open biopsy forceps measuring 6 mm.

Monitored anesthesia care (MAC) was used for all dilation sessions except for combined antegrade-retrograde dilations (CARD), which required general anesthesia. Size of stenosis was estimated based on the diameters of the endoscopes used, which were either Olympus® 5.4 mm GIF-XP180N, 5.5 mm GIF-XP190N (Figure 1), 9.2 mm GIF-H190, or 9.8 mm GIF-180 endoscopes. Pediatric open biopsy forceps (six millimeters end-to-end) were also used to estimate the stenosis diameter (Figure 2). Wire-guided Diversatek Medovations™ Savary bougie dilators were used for dilation in all patients. All patients were severely symptomatic ranging from aphagia to dysphagia with small amounts of soft foods. Dilation sessions were scheduled every two to four weeks at the start of dilation therapy and according to the severity of stenosis thereafter. The stenosis diameter was measured at the beginning of the first dilation session and at the beginning of each dilation session thereafter. The maximum caliber of the dilator that was passed was recorded for each dilation session. Dilators of a progressively increasing caliber were passed with each successive dilation (i.e., increase of 1-2 mm dilator sizes with each dilation) until encountering severe resistance (18 mm caliber (54 Fr) if possible). Dilation was performed in all cases by an experienced endoscopist. In two patients for a total of five dilations (patients #2 and #4), endoscopic injection of 40 mg of triamcinolone acetonide into the stricture was performed. The decision to inject triamcinolone was based on endoscopist experience.

**Figure 3:**
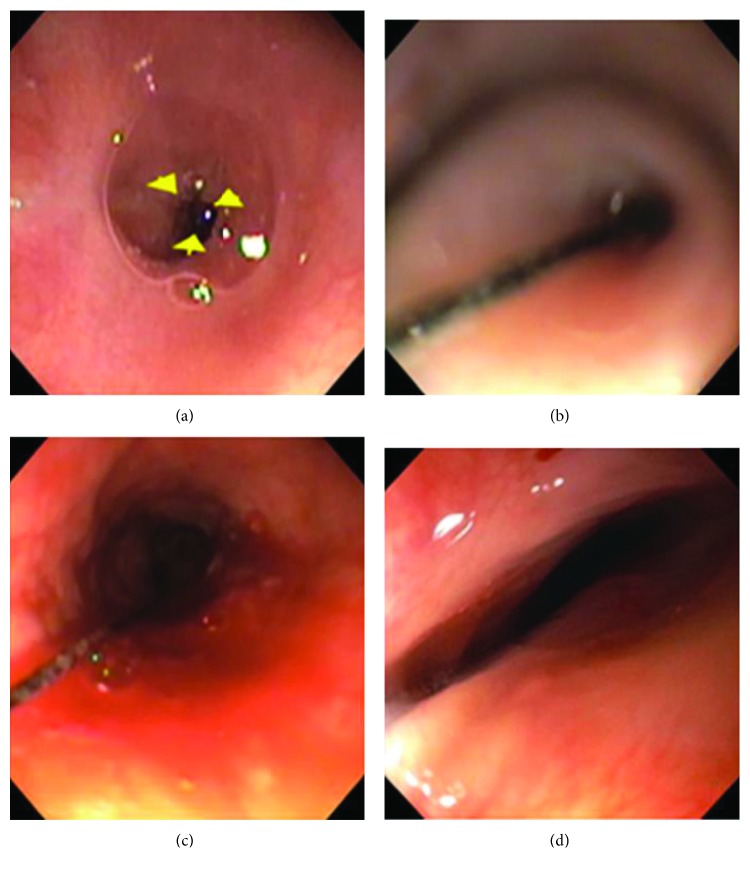
(a–d) All images from the same patient, taken using 5 mm XP scope. (a) Retrograde view of the stricture at the first dilation, opening approximately 2 mm. (b) Antegrade view of the stricture 3 weeks later with interval placement of a lumen patency string, opening approximately 2 mm. (c) Antegrade view of the stricture 3 weeks later postdilation, the largest dilator passed 9 mm in diameter (27 Fr), a string in place. (d) Antegrade view of the stricture approximately 1 year later, predilation, proximal end measuring 8 mm in diameter × 2 cm in length, a lumen patency string no longer present.

All patients underwent anterograde dilation for most of their dilations; however, 5/7 (71%) patients had strictures at presentation that required a retrograde approach to dilate. This was required when the 5.4 or 5.5 mm endoscope could not be advanced antegrade. The initial procedure involves combined antegrade-retrograde dilation (CARD) in which a thin (pediatric size) endoscope is passed retrograde via the gastrostomy stoma while a standard-caliber endoscope is passed antegrade through the oropharynx [13]. If necessary, the gastrostomy stoma tract is dilated to allow passage of the retrograde endoscope [14]. Both endoscopes are carefully impacted into their respective blind ends, and a guidewire is passed beyond the level of the retrograde endoscope. Using a combination of air insufflation, transillumination, and careful wire probing, a thin semilucent membrane is identified and penetrated and the stricture is traversed by the guidewire [15]. Direct visualization with the antegrade scope is vital to minimize the risk of perforation. The use of triplane (anterior-posterior, lateral, and oblique) fluoroscopy with the use of a C-arm is frequently needed (as was the case in four of our five patients needing retrograde dilation) to ensure proper antegrade-retrograde axis alignment [13]. Once the guidewire is visualized by the antegrade scope, it is grasped and pulled out of the patient's mouth. A series of sequentially larger Savary dilators is then passed retrograde (from the gastrostomy stoma through the mouth) until moderate to severe resistance is reached. Because of a concern for complete lumen closure in between dilation procedures, a synthetic multibraid, high-tensile strength string (called the lumen patency string) is attached to the guidewire that traverses the newly restored lumen and pulled retrograde through the mouth. Once recovered from the mouth, the proximal end of the string is retrieved via a nostril, tied to the distal end of the string (out of the gastrostomy stoma), and secured onto the upper anterior chest wall [13, 16]. For future dilation sessions, Savary dilators are attached to the distal end of the string and pulled retrograde across the path of the string. See Table 2 for dilation characteristics and Figures 3(a)–3(d) for an example of the chronological progression of a stricture treated with serial dilations.

### 2.3. Definitions of Variables

Traditionally, a benign esophageal stricture is considered refractory if luminal patency of ≥14 mm could not be achieved after ≥5 dilation sessions at intervals of two to four weeks [10]. The same authors [9, 10] define achievement of technical success as the ability to dilate a stricture at least three mm during initial dilation therapy. We used the above definitions of these terms in our study. For our group of patients, we defined clinical success as the ability to advance diet consistency (i.e., from liquids to solids) from baseline diet at start of the study period and the need for repeat dilation being no fewer than every 12 weeks. Complex strictures were defined as long (>2 cm) or associated with a diameter of 10 mm or less.

### 2.4. Diet Outcomes

Determination of dysphagia improvement and diet advancement was made by chart review of documentation by SLP and/or the endoscopist descriptive notes throughout the study period. The need for a G-tube to maintain optimal nutritional status was assessed at the beginning and again at the end of the study period based on electronic medical record chart review. In the same fashion, the best tolerated oral diet was determined at the beginning and again at end of the study period. Diet was described in five categories: NPO, liquid, pureed, soft, or regular diet. Chapuy et al.'s [17] Diet/GT scale was used as a measure of diet advancement and surrogate measure for clinical success of dilation therapy (see Table 2). This scoring system combines GT status and diet and is as follows: Score 1, GT present and NPO; Score 2, GT present and liquid/pureed diet; Score 3, GT present and soft/regular diet; Score 4, no GT and liquid/pureed diet; and Score 5, no GT and soft/regular diet.

## 3. Results

### 3.1. Dilation Outcomes

Seven patients underwent a total of 118 dilations (see Table 2). All strictures were complex. Of the 6 patients who had TEPs placed, TEP displacement during dilation occurred in only 2 patients a total of 4 times out of 106 dilations. There were no hospital admissions for TEP displacements and no further sequela. There were no tumor recurrences found during any of the dilation procedures. The median number of dilations per patient was 12 (range 7 to 48), and the median follow-up was 11 months. The average interval in between dilations was six weeks. Median time from laryngectomy to end of the study period was 212 weeks (53 months) and varied considerably (range 40 to 709 weeks), but did not seem to effect technical success, clinical success, or changes in Diet/GT score.

Technical success was achieved in 6/7 patients (85.7%), and clinical success was achieved in 2/7 patients (28.6%). Average diameter of esophageal stenosis at time of the first dilation was 4.3 mm (median 3 mm), with one complete lumen occlusion. The maximum caliber dilator passed was 18 mm (54 Fr) in 6/7 patients (16 mm, or 48 Fr, in patient #3). A retrograde approach was used in 5/7 (71%) patients in a total of 25 dilations. Fluoroscopic guidance was needed in 4/7 patients (57%). A lumen patency string was used in four patients. The average time the string was kept in place was 26 weeks (range 10 to 45 weeks). Intralesional steroids were used in two patients in a total of four occasions.

### 3.2. Diet Outcomes

Prior to the first dilation, five of seven patients had gastrostomy tubes (GT) for enteral feeding. Two patients (patients #5 and #7) in whom GTs were deemed beneficial repeatedly refused GT placement. At study end, all five patients who were GT-dependent at start of the study still had their GT in; however, three were not using their GT at all and four were tolerating a soft-regular diet. All patients except patient #1 (six out of seven) had improvements in their Diet/GT scores (see Table 2).

## 4. Discussion

Rates of pharyngoesophageal stricture formation after laryngectomy range from 13 to 50% [1]. Studies investigating whether the type of primary pharyngeal reconstruction after total laryngectomy affects the risk of stricture formation have yielded mixed results [1, 18–21]. In addition, while surgical risk factors for the development of recurrent and refractory strictures after esophagectomy have been described in the literature [9], surgical risk factors for the development of recurrent or refractory PLS are not well reported. In their studies, Sweeny et al. [1] and Walton et al. [21] found that PLS required “serial dilations” in 45% and 33% of cases, respectively. However, data on the exact number of dilations required, intervals at which dilations were done, presence of dysphagia, and endoscopic findings were not reported in these studies, so it is unknown what percentage of these strictures (if any) were truly recurrent and/or refractory.

Pharyngocutaneous fistula (PCF) is a well-known early complication associated with TL. Salvage laryngectomy, previous chemoradiation, low albumin, and pharynx reconstruction are known risk factors for PCF development [22, 23]. PCF can be treated with conservative measures in nonchemoradiated patients, whereas adjuvant hyperbaric oxygen therapy and/or surgical closure (i.e., flap coverage) are indicated for patients with prior chemoradiation [24]. Salivary bypass tube (SBT) is also commonly placed following TL for PCF prevention or employed at time of surgical repair of the PCF. Long-standing PCF can predispose to neopharyngeal stricture formation [25], although this complication is rare [26]. This association is also not well reported in the literature. Three of our patients had PCF, and an additional patient had an orocutaneous fistula. In all cases, fistulae were successfully treated yet patients went on to develop refractory strictures. This raises the question whether PCF, even after it has resolved, could be a potential risk factor for the development of refractory PLS. Further larger comparative studies are needed to investigate this potential association.

Studies investigating clinical or endoscopy-based features associated with the development of refractory PLS are also lacking. It is generally recognized that complex strictures tend to be refractory to dilation [27]. In addition, endoscopic findings of cicatricial luminal compromise or fibrosis in the absence of inflammation often point to strictures that will be refractory [9, 10, 28]. In efforts to further define additional endoscopic risk factors, Mendelson et al. [9] studied 74 patients with esophageal anastomotic strictures (46 postesophagectomy and 28 postlaryngectomy). They found that the need for the use of fluoroscopy (needed in four of our seven patients) during dilation was associated with the development of refractory benign strictures. In our study, fluoroscopic guidance was used in four of seven patients. Interestingly, while chemotherapy is associated with increased incidence of strictures, they found that neoadjuvant chemotherapy was associated with a reduced risk of a stricture being refractory to dilation. Race, prior radiation therapy, type of cancer, age, and removal of staples or a suture material were not significant predictors associated with refractory strictures in their study.

The precise location of PLS in relation to the inferior pharyngeal constrictor muscles, the anatomical cervical esophagus, or the cricopharyngeus is difficult to delineate. In our experience, regardless of the exact location (i.e., neopharynx vs. proximal cervical esophagus), the proximal nature of PLS may predict strictures of a more refractory nature that are more technically difficult to dilate. In addition, postlaryngectomy patients may have a tracheoesophageal prosthesis (TEP) in place (six of our seven patients did), with which there is an additional technical risk of TEP displacement during dilation. While it did not reach statistical significance (*p* = 0.07), rates of refractory strictures were higher in postlaryngectomy patients as compared to postesophagectomy patients in the above study by Mendelson et al. [9].

While self-expanding metal stents (SEMS) have been used in the treatment of refractory benign esophageal strictures, we do not believe that there is a role for SEMS use in the treatment of PLS. The surgical site has impaired motility and lacks muscular coordination. The proximal stent (top) protrudes at least one cm above the stricture opening to prevent migration and will impair food bolus passage. In addition, patients frequently complain of a globus sensation. Stent migration is also an issue, with a migration rate of 28.6% in refractory esophageal benign strictures (RBES) reported in a recent meta-analysis of 444 patients by Fuccio et al. [29]. Stent placement also carries a risk of several life-threatening complications, including immediate respiratory compromise, aspiration, fistula formation, sepsis, and even hemorrhage from stent erosion into the esophageal wall or carotid artery [30, 31].

Patients with high-grade, refractory/recurrent strictures frequently require a GT to maintain enteral nutrition. Not surprisingly, five of the seven patients in our study required GTs, and GT was recommended for the additional two patients but was refused. In our experience, we do not want to remove the GT unless we are sure that the patient will not need it again.

There were no reported complications in our study aside from self-limited bleeding. Minimal complication rates have been reported in prior endoscopic dilation series [4, 7, 9–11, 32, 33], confirming the overall safety of endoscopic dilation for the treatment of pharyngoesophageal strictures. Our retrospective study had several limitations, most notably the small number of patients studied. This was due to the highly selected patient population at a single institution over just a 4.5-year period. As patients came to our center from multiple outside institutions, there were inherent difficulties and inconsistencies in obtaining all pertinent data about these patients. The dose of radiation and technique of radiation therapy used (conventional vs. intensity-modulated radiation therapy [IMRT]) were not available for some of our patients. In addition, dysphagia assessment in conjunction with a speech pathologist was not performed in all patients. Modified barium swallow (MBS) was used at least once in 6/7 patients, but the timing of MBS was highly variable (i.e., before the first dilation vs. midway through dilations in the study period). A few of our patients did not have their laryngectomies at our institution and therefore information regarding the amount of initial mucosal preservation is lacking. Finally, some may have had dilations prior to the ones recorded in this study at our institution, which may have affected our results.

Endoscopic dilation therapy requires strong commitment and patient adherence, as evidenced by the frequent number of dilation sessions (median number of dilations, 12) during an extended period of time (median follow-up, 11 months) seen in our series. Unfortunately, the very need for repeated dilations is not surprisingly associated with an increased risk of persistent dysphagia [1, 33]. Nevertheless, adherence to frequent dilations can lead to relief of dysphagia, as evidenced by the ability of patient #6 to advance his/her diet and an improved quality of life. The ability to remove and/or not be dependent on a GT is invaluable in these patients. In a study by Terrell et al. [34] that measured quality of life in 570 patients with head and neck cancer, the presence of a feeding tube had the most negative impact on quality of life of all studied factors, which included medical comorbid conditions, presence of a tracheostomy tube, chemotherapy, and neck dissection. All patients at the end of the study indicated that the inconveniences and resources expended during dilation therapy were well worth their subjective improvement in dysphagia relief. Therefore, if patients are willing to continue, patient adherence to serial dilations is key to the relief of dysphagia.

Our clinical success rate was only 28.6% despite 6/7 patients (85.7%) having advancements in their diets and subjective improvement of dysphagia. This was because the second part of our proposed definition of clinical success required the need for repeat dilation to be no fewer than every 12 weeks. Similarly, in their study of refractory benign esophageal strictures, Repici et al. [35] defined clinical success as “no need for endoscopic interventions for at least 6 months.” Mendelson et al. [9] defined clinical success as “resolution of dysphagia and achieving luminal patency for ≥1 month.” Given this need for serial dilations to relieve dysphagia and improve oral intake that was demonstrated in our study (and others), we propose that any definition of clinical success in patients with refractory PLS avoid the use of a specific time interval between dilations to characterize success. As an alternative, we propose that future studies focus on measures of functional success of dilation, specifically focusing on diet and GT status as was done by Chapuy et al. [33].

## 5. Conclusion

Serial endoscopic dilation therapy at regular, short-time intervals is required in the treatment of refractory PLS in order to maintain luminal patency, relieve dysphagia, and advance a patient's best tolerated diet. Dilation of these strictures is safe and effective and frequently requires the use of a retrograde approach (rarely CARD), fluoroscopic guidance, and/or lumen patency strings. Given this, the need arises to modify the definition of clinical success in these patients, specifically avoiding the use of a specific time interval between dilations to characterize success. Due to the small number of patients in our study, risk factors associated with the development of refractory PLS could not be appropriately identified. Future studies using prospectively collected data on the postlaryngectomy patient population, including prior chemotherapy or radiation, TL complications (i.e., PCF), and precise stricture location, can determine occurrence rates of refractory PLS and lead to the identification of risk factors and the standardization of a treatment approach in these patients.

## Figures and Tables

**Table 1:** Patient and total laryngectomy characteristics.

Patient	Age	Sex	Malignancy and staging	Malignancy treatment	TL indication	TL surgical approach	TEP placement?	Postop complications (other than strictures)	Salivary bypass tubes?
#1	60	M	SCC of the left supraglottic larynx (T4aN2cM0)	Primary TL with adjuvant postop chemoRT (cisplatin)	Treatment of malignancy	TL, partial pharyngectomy, partial glossectomy, tracheoesophageal puncture, cricopharyngeal myotomy, partial left thyroidectomy with right sternocleidomastoid muscle pedicle rotational flap and left radial forearm free flap	Yes (primary)	PCF; leakage around TEP; 2 inadvertent TEP dislodgements (replaced)	No
#2	69	F	Cancer of epiglottis with the type and stage unknown; primary SCC of the left supraglottic larynx (unknown stage) 27 years later	Surgical resection and postop XRT of epiglottic cancer; primary chemoRT (unsure cancer stage, radiation dose or type of chemo) for primary laryngeal cancer	Nonfunctional larynx with chronic aspiration and significant dysphagia	TL, partial pharyngectomy, right sternocleidomastoid muscle pedicle flaps, left pec muscle flap	Yes (secondary)	None	No
#3	58	F	SCC of the glottic larynx (T3N2bM0), later restaged to T4N0M0	ChemoRT initially, then primary TL with adjuvant postop chemoRT (cisplatin)	Treatment of malignancy	TL, left modified radical neck dissection, primary TP puncture and cricopharyngeal myotomy	Yes (primary)	Orocutaneous fistula with chyle leak; leakage around TEP; 2 TEP embeddings (resolved with outpatient speech language pathologist intervention)	Yes
#4	58	M	SCC of the larynx (unknown stage)	ChemoRT, developed recurrent disease 1 year later and required salvage laryngopharynectomy	Treatment of malignancy	Laryngopharyngectomy with left pectoral flap and bilateral neck LN dissection	No	PCF; extensive peristomal granulation tissue s/p surgical excision; bleeding from a stoma	No
#5	61	M	SCC of the larynx (T4N0M0), incidental papillary thyroid cancer	Primary TL and right hemithyroidectomy with adjuvant postop chemoRT (cisplatin and 70 Gy)	Treatment of malignancy	TL, right hemithyroidectomy, tracheoesophageal puncture, cricopharyngeal myotomy	Yes (primary)	None	No
#6	50	F	SCC of left TVC (T3N2bM0)	XRT and left modified radical neck dissection ~25 years ago	Chondroradionecrosis of the laryngeal structures and laryngocutaneous fistula	TL with left pectoral flap	Yes (secondary)	PCF; 20 hyperbaric treatments	Yes
#7	64	M	SCC of the larynx (T3N1M0)	Primary TL with adjuvant postop XRT	Treatment of malignancy	TL with left neck dissection	Yes (secondary)	Perforation of the posterior cervical esophageal wall after secondary TP requiring gastrostomy and hyperbaric O_2_ at outside facility; leakage around TEP; Candida overgrowth of a stoma	No

**Table 2:** Dilation characteristics and measures of success.

Patient	Size of stenosis at time of the first dilation (diameter in mm)	Max luminal diameter achieved by study end (mm)^∗∗^	Time from TL to the first dilation (weeks)	Time from TL at study end (weeks)	Number of dilations at study end	Average time interval between dilations (weeks)	Retrograde approach used?	Lumen patency string in place (weeks)	Use of fluoroscopy	Diet/GT score^∗∗∗^: start of the study period	Diet/GT score: end of the study period	Technical success?	Clinical success?
#1	5	10	32	40	12	3.9	Yes	10	No	2	2	Yes	No
#2	2	6	36	85	14	3.8	Yes	39	Yes	2	3	Yes	No
#3	2	6	255	338	15	6	Yes	n/a	Yes	2	3	Yes	No
#4	3	6	53	102	12	4.5	Yes	10	Yes	2	3	Yes	No
#5	10	12	95	212	10	13	No	n/a	No	4^∗∗∗∗^	5	Yes	Yes
#6	0 (CLO^∗^)	11	58	237	48	3.5	Yes	45	Yes	1	5	No	No
#7	8	9	661	709	7	8.5	No	n/a	No	4^∗∗∗∗^	5	Yes	Yes

^∗^Complete lumen occlusion. ^∗∗^Max luminal caliber achieved prior to dilation during the study period. ^∗∗∗^Diet/GT scale: Score 1 = GT present and NPO, Score 2 = GT present and liquid/pureed diet, Score 3 = GT present and soft/regular diet, Score 4 = no GT and liquid/pureed diet, and Score 5 = no GT and soft/regular diet. ^∗∗∗∗^GT recommended but patient refused.

## Data Availability

The retrospective data used to support our findings in this study was gathered from the University of Florida Integrated Data Repository (https://www.ctsi.ufl.edu/research-initiatives/completed-projects/integrated-data-repository/) and deposited in the University of Florida REDCap (Research Electronic Data Capture) database. We would like to acknowledge April A. Goddard and Tiffany D. Harrison for helping with IRB preparation and submission.
